# Single crystal X-ray structural dataset of 1,2,4-dithiazolium tetrafluoroborate

**DOI:** 10.1016/j.dib.2022.107924

**Published:** 2022-02-04

**Authors:** Balasubramaniam Arul Prakasam, Chandran Udhaya Kumar, Manu Lahtinen, Anssi Peuronen, Mika Sillanpää

**Affiliations:** a Department of Chemistry, Annamalai University, Annamalainagar, 608002, India; b Department of Chemistry, P.O.Box 35, FI-40014 University of Jyväskylä, Jyväskylä, Finland; c Group of Intelligent Materials Chemistry, Department of Chemistry, University of Turku, FI-20014 Turku, Finland; d Department of Chemical Engineering, School of Mining, Metallurgy and Chemical Engineering, University of Johannesburg, P. O. Box 17011, Doornfontein 2028, South Africa; e Chemistry Department, College of Science, King Saud University, Riyadh 11451, Saudi Arabia; f Faculty of Science and Technology, School of Applied Physics, University Kebangsaan Malaysia, 43600 Bangi, Selangor, Malaysia; g Department of Biological and Chemical Engineering, Aarhus University, Nørrebrogade 44, 8000 Aarhus C, Denmark

**Keywords:** 1,2,4-Dithiazolium, Crystal structure, Hydrogen bond, Heterocycle

## Abstract

Herein, we present the crystallographic dataset of 1,2,4-dithiazolium tetrafluoroborate. Single crystal X-ray structural analysis evidences that the 1,2,4-dithiazolium ring is almost planar. The 1,2,4-dithiazolium and tetrafluoroborate ions contribute in hydrogen bonding wherein the N-H·N hydrogen bonding in 1,2,4-dithiazolium dimer forms an eight-membered pseudo ring with the $R_{2}^{2}\left(8\right)$ Etter's graph set. The information provided in this data contributes to the understanding of structural chemistry and hydrogen bonding interactions in dithiazole derivatives.

## Specifications Table

**Table:** 

Subject	Organic Chemistry
Specific subject area	Organic heterocyclic molecules and crystallographic structure
Type of data	Figures and Tables
How data were acquired	Single Crystal X-ray diffraction: Agilent Supernova (Cu/Mo dual flux micro-focus sources) diffractometer.
Data format	Raw data and Analyzed
Parameters for data collection	SXRD: Agilent Supernova (Cu/Mo dual flux micro-focus sources) diffractometer was used to acquire the data at 123 K using Cu-K_α_ radiation (λ= 1.54184 Å).
Data source location	Department of Chemistry, Laboratories of Inorganic and Analytical Chemistry, University of Jyväskylä, Finland.
Data accessibility	The data can be accessed at https://data.mendeley.com/datasets/6zw4w4kvsc/1 or from the Cambridge Crystallographic Data Centre. CCDC No. 2109451. Copies of the data can be obtained free of charge via http://www.ccdc.cam.ac.uk/conts/retrieving.html, or from the Cambridge Crystallographic Data Centre, 12 Union Road, Cambridge CB2 1EZ, UK; fax: +44 1223 336 033: or e-mail: deposit@ccdc.cam.ac.uk.

## Value of the Data

• The presented data will be useful for the organic chemists and structural chemists.
• The data will be useful to analyze the hydrogen bonding pattern in crystal structures of dithiazolium salts.
• The data may be useful for the comparison of aromatic character of the heterocyclic cations.
• The data may provide information pertaining to single crystal X-ray structural analysis of ionic liquids.

## 1 Data Description

**Fig. 1:**
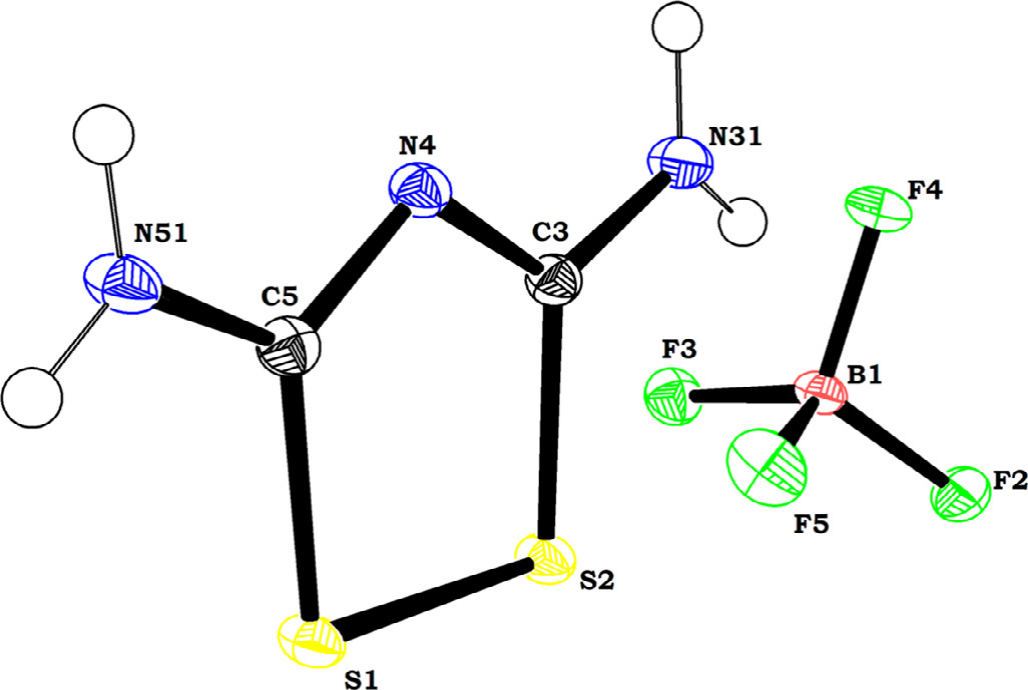
ORTEP image of 1,2,4-dithiazolium tetrafluoroborate.

**Fig. 2:**
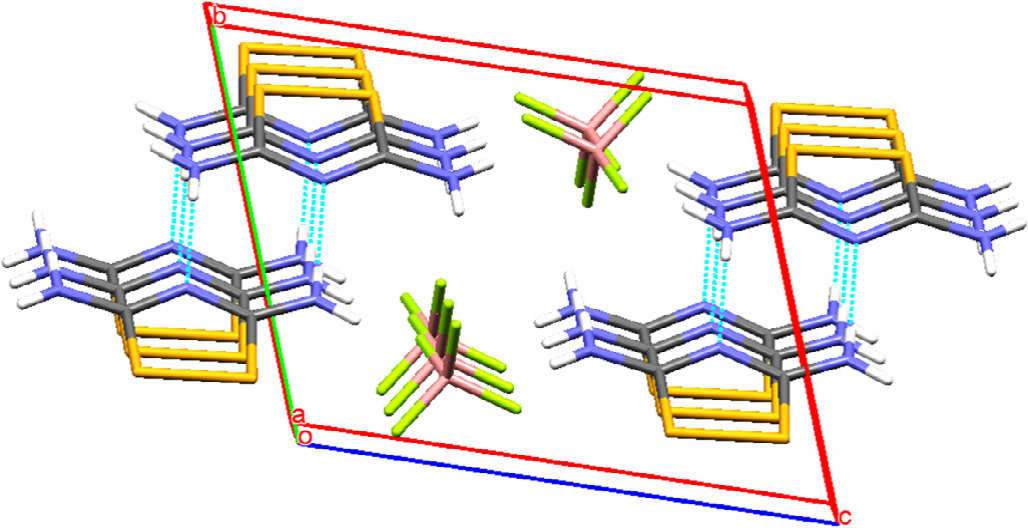
Packing diagram of 1,2,4-dithiazolium tetrafluoroborate.

This work describes the data with respect to the new crystal structure of 1,2,4-dithiazolium tetrafluoroborate. ORTEP drawing of 1,2,4-dithiazolium tetrafluoroborate is shown in Fig. 1 and the packing diagram is shown in Fig. 2. There are two molecules in the unit cell and the 1,2,4-dithiazolium ring is planar. The amino substituents at carbon atoms 3 and 5 are coplanar with the central ring. The bond lengths suggest that there is a delocalization of π electrons in the N–C–N–C–N fragment. Nevertheless, the dithiazolium ring is devoid of aromatic character as there is no delocalization of π electrons throughout the ring. The 1,2,4-dithiazolium and tetrafluoroborate ions contributed in hydrogen bonding wherein, the N-H·N hydrogen bonding in 1,2,4-dithiazolium dimer forms an eight membered pseudo ring with the $R_{2}^{2}\;\left(8\right)$, Etter's graph set [1]. The details of crystal data with structure refinement [[2], [3], [4]], bond distances, bond angles, torsional angles, fractional atomic coordinates, anisotropic atomic displacement and hydrogen bonds are depicted in Table 1, Table 2, Table 3, Table 4, Table 5, Table 6, Table 7 respectively. The data deposited with the repository Mendeley and can be accessed at https://data.mendeley.com/datasets/6zw4w4kvsc/1 [5].

**Table 1:** Crystal data and structure refinement of 1,2,4-dithiazolium tetrafluoroborate.

Empirical formula	C_2_H_4_BF_4_N_3_S_2_
FW Crystal dimensions (mm)	221.01 0.167 × 0.1244 × 0.072
Crystal system	triclinic
Color	pale yellow
Space group	*P*-1
a/Å	5.2098(10)
b/Å	7.7233(15)
c/Å	9.742(16)
α/°	110.2(16)
β/°	90.664(15)
γ/°	93.314(16)
Volume/ Å^3^	367.06(12)
Z	2
Dc/gcm^−3^	2.000
μ/cm^−1^	6.906
F(000)	220
2θ range/°	9.68 to 137.954
Index ranges	-6≤ *h* ≤ 6, -9 ≤ *k* ≤ 6, -9 ≤ *l* ≤ 11
Reflections collected	2129
Observed reflections F_0_>4σ(F_0_)	1353
Number of parameters refined	122
Final R, R_w_ (obs, data)	0.0234, 0.0588
GOOF	1.051

**Table 2:** Selected bond distances (Å) for 1,2,4-dithiazolium tetrafluoroborate.

S1–S2	2.0586(6)
S1–C5	1.7507(16)
S2–C3	1.7535(16)
F3–B1	1.40(2)
F4–B1	1.4005(19)
F5–B1	1.389(2)
F2–B1	1.385(2)
N31–C3	1.309(2)
N51–C5	1.312(2)
N4–C5	1.334(2)
N4–C3	1.337(2)

**Table 3:** Selected bond angles (°) for 1,2,4-dithiazolium tetrafluoroborate.

C5–S1–S2	92.86(5)
C3–S2–S1	93.29(6)
C5–N4–C3	114.53(14)
N51–C5–S1	118.30(12)
N51–C5–N4	121.73(15)
N4–C5–S1	119.96(12)
N31–C3–S2	118.77(12)
N31–C3–N4	121.93(15)
N4–C3–S2	119.30(12)

**Table 4:** Selected torsional angles (°) for 1,2,4-dithiazolium tetrafluoroborate.

S1–S2–C3–N31	177.60(12)
S1–S2–C3–N4	−2.46(12)
S2–S1–C5–N51	179.02(12)
S2–S1–C5–N4	−0.11(12)
C5–N4–C3–S2	2.80(18)
C5–N4–C3–N31	−177.26(13)
C3–N4–C5–S1	−1.54(18)
C3–N4–C5–N51	179.35(13)

**Table 5:** Fractional Atomic Coordinates (× 10^4^) and Equivalent Isotropic Displacement Parameters (Å^2^ × 10^3^) for 1,2,4-dithiazolium tetrafluoroborate. U_eq_ is defined as 1/3 of of the trace of the orthogonalised U_IJ_ tensor.

Atom	*x*	*y*	*Z*	U(eq)
S1	3695.4(7)	1724.9(5)	9321.2(4)	12.94(13)
S2	3281.5(7)	1482.7(5)	7157.3(4)	12.81(14)
F3	28.5(18)	3487.9(13)	3468.8(10)	18.8(2)
F4	4125.4(19)	2802.7(14)	2769.0(11)	23.5(2)
F5	2170(2)	1544.7(15)	4304.1(11)	25.1(3)
F2	820.6(19)	609.1(13)	1918.0(10)	21.2(2)
N31	6895(3)	3037(2)	5994.1(15)	15.2(3)
N51	7706(3)	3712(2)	10875.9(15)	14.6(3)
N4	7524(2)	3540.4(18)	8460.3(14)	12.1(3)
C5	6567(3)	3108(2)	9573.0(16)	12.0(3)
C3	6155(3)	2802(2)	7201.0(17)	11.8(3)
B1	1774(3)	2101(2)	3109.8(18)	13.4(3)

**Table 6:** Anisotropic atomic displacement parameters (Å^2^ × 10^3^) for 1,2,4-dithiazolium tetrafluoroborate.

Atom	U_11_	U_22_	U_33_	U_23_	U_13_	U_12_
S1	10.2(2)	17.4(2)	11.8(2)	6.37(15)	−0.23(13)	−2.78(14)
S2	10.4(2)	16.4(2)	11.0(2)	4.60(15)	−0.82(13)	−2.91(14)
F3	14.8(5)	21.7(5)	19.5(5)	6.5(4)	−0.1(4)	1.7(4)
F4	14.2(5)	24.2(5)	29.2(5)	6.4(4)	6.2(4)	−5.3(4)
F5	25.5(5)	35.6(6)	19.5(5)	16.1(5)	−1.9(4)	3.5(4)
F2	20.6(5)	20.5(5)	17.3(5)	1.1(4)	−0.9(4)	−5.8(4)
N31	14.5(7)	18.6(7)	12.2(6)	5.6(6)	−0.3(5)	−4.4(6)
N51	10.7(6)	21.6(7)	12.5(6)	8.0(6)	−1.3(5)	−3.8(5)
N4	9.0(6)	14.5(6)	12.6(6)	4.6(5)	−0.2(5)	−0.5(5)
C5	9.4(7)	12.4(7)	14.3(7)	4.7(6)	0.2(5)	1.7(6)
C3	9.5(7)	11.4(7)	14.2(7)	3.9(6)	0.9(5)	0.6(5)
B1	10.8(8)	17.4(8)	11.8(8)	5.5(7)	0.3(6)	−2.7(6)

**Table 7:** Hydrogen bonds for 1,2,4-dithiazolium tetrafluoroborate.

D	H	A	d(D-H)/Å	d(H-A)/Å	d(D-A)/Å	D-H-A/°
N31	H31A	F3^1^	0.85(2)	2.15(2)	2.9258(19)	151.1(19)
N51	H51A	F4^2^	0.84(2)	2.08(2)	2.8631(17)	155.7(19)
N51	H51B	N4^3^	0.81(2)	2.28(2)	3.082(2)	172(2)
N31	H31B	F5	0.84(2)	2.14(2)	2.8926(19)	148.0(19)

1 1-X,1-Y,1-Z;

2 +X,+Y,1+Z;

3 2-X,1-Y,2-Z

## 2 Experimental Design, Materials and Methods

### 2.1 Synthesis of 1,2,4-dithiazolium tetrafluoroborate

The title compound was prepared by refluxing a mixture of dithiobiuret (1 mmol) and BF_3_:Et_2_O (1.2 mmol) in 95% ethanol for a period of 12h. The reaction mixture was then filtered and left to evaporate. After two days, the solid obtained was recrystallized from dichloromethane:methanol (1:1) solvent mixture.

### 2.2 Single crystal X-ray structural analysis

Single crystals suitable for X-ray structural analysis were obtained by slow evaporation using dichloromethane:methanol (1:1) solvent mixture. Single crystal dataset used in structure determination was acquired with dual source (Cu/Mo) Agilent SuperNova diffractometer equipped with multilayer optics for generating monochromatized Cu K_α_ radiation, and Atlas CCD detector for recording data. A crystal was mounted in a MiTeGen MicroMount™ loop (100 μm), and data collection was made at -150°C under N_2_ stream. Data collection, reduction and analytical numeric absorption corrections by multifaceted crystal models were all made using CrysAlisPRO program [2]. By using Olex^2^ (v 1.3) [3], the crystal structure was solved with Superflip and refined on *F^2^* by full matrix least squares techniques with ShelXL [4] program. All non-hydrogen atoms were refined with anisotropic displacement parameters, whereas hydrogen atoms were located from the electron density map and refined freely except of using isotropic displacement parameters 1.2 times of the host atom.

## Ethics Statement

The work was not involved with human subjects or animal experiments and the data was not collected from social media platforms.

## CRediT Author Statement

**Balasubramaniam Arul Prakasam:** Conceptualization, Investigation, Methodology, Writing – Original Draft; **Chandran Udhaya Kumar:** Data Curation, Writing – Review & Editing; **Manu Lahtinen:** Investigation, Data Curation, Validation, Writing - Review & Editing. **Anssi Peuronen:** Investigation, Data Curation, Validation, Writing – Review & Editing; **Mika Sillanpää:** Methodology, Validation.

## Declaration of Competing Interest

The authors state that they have no known competing financial interests or personal relationships that could have appeared to influence of the work reported in this paper.

## Appendix Supplementary materials

Image, application 1

Image, application 2
